# Primary syphilis without chancre – A case report of rare syphilitic balanitis of Follmann

**DOI:** 10.3389/fmed.2022.958456

**Published:** 2022-09-21

**Authors:** Xu-qi Ren, Qun-ling Nie, An-qi Liu

**Affiliations:** Department of Dermatology, Affiliated Hospital of Nantong University, Nantong, China

**Keywords:** syphilis, primary, primary syphilis without chancre, syphilitic balanitis, rare

## Abstract

**Introduction:**

Syphilitic balanitis of Follmann (SBF) is a rare condition of primary syphilis which is characterized by any kind of balanitis with or without chancre on the penis combined with the presence of swollen inguinal lymph nodes confirmed by the finding of Treponema pallidum in the lesions or by the positive serological syphilitic testing. Timely identification of the SBF is very important in properly treating the disease stopping the spread of syphilis.

**Case presentation:**

A 42year-old heterosexual male patient came to our clinic and complained of a painless, hard erythema nodule with a whitish scale in his coronal sulcus of the penis for about a week. The dermatologic examination revealed an infiltrative, hard erythematous lesion surrounding the coronal sulcus of the patient’s penis, with mild erosion and a small amount of exudation. There was a whitish pseudomembrane-like covering on the surface of the erythematous lesion in the coronal sulcus, which is mimicked as candidal balanitis. The result of the fungus microscopic examination was negative, while the laboratory findings showed positive results in serologic syphilitic testing. The patient was diagnosed with primary syphilis and intramuscularly treated with a dose of benzylpenicillin of 2.4 million units. The patient’s skin lesions disappeared completely 60 days after penicillin treatment.

**Conclusion:**

To our knowledge, this is the first SBF case reported in China. Syphilitic balanitis of Follmann may have variable clinical appearances. We emphasize that when balanitis with risky sexual activities or with sexually transmitted diseases, the diagnosis of SBF should be kept in mind.

## Introduction

The incidence of syphilis has been increasing steadily in recent years (1, 2). It is believed that the agent of syphilis, *Treponema pallidum* (TP), invades the human skin or mucosa, then spreads to our body, and forms a long-term persistent chronic infection (3). Based on the time of infection, syphilis was divided into two stages, early and late stages. Early syphilis includes primary, secondary, and early latent syphilis. Among those, primary and secondary syphilis are very contagious. Therefore, it is very important to treat the infected people in the early stages to stop the spread of the disease.

The syphilitic chancre is usually described as a painless, solitary, superficially eroded, genitalia involved, and self-healing erythematous nodular lesion (4). Although the typical syphilitic chancres are easy to identify, a low proportion of incident, clinical primary, and secondary syphilis cases are reported in the early stage, and in the United States, only 32% of primary and secondary syphilis cases were identified in the primary stage (5, 6). The fact suggests that a considerable number of primary syphilis cases are misdiagnosed or missed, which will greatly increase the spread of the disease. One of the reasons for the misdiagnosis of primary syphilis is the “mysterious character” of syphilitic chancers (7, 8). Herein, we report a case of primary syphilis with mucocutaneous manifestations mimicking candida balanitis, without obvious chancre, a rare manifestation in primary syphilis, so-called “syphilitic balanitis of Follmann” (SBF).

## Case presentation

On 8th March 2022, a 42-year-old male patient came to our clinic and complained that he had a skin lesion on his penis for nearly a week and reported that he found a hard, strip of erythema distributed in the coronal sulcus on the left side of the penis at the beginning, and then quickly covered with the whitish substance on the surface of the erythema. Thereafter, the skin lesion gradually enlarged to a circle around the coronal sulcus. The lesion was painless with a mild burning sensation.

The patient is heterosexual and had his first sexual activity at the age of seventeen and divorced 4 years ago. He also reported that he had a history of taking methamphetamine for more than 10 years and was treated in Yixing’s compulsory isolation drug treatment center in Jiangsu Province, China from October 2018 to December 2020. There was no relapse after drug treatment for more than 1 year. His medical history was generally normal except that for hypertension and he had no erosion or ulcer or blister on his penis before. When he was in the drug rehabilitation center, he underwent sexually transmitted diseases (STDs) testing, including Hepatitis B Virus (HBV), Hepatitis C Virus (HCV), Human Immunodeficiency Virus (HIV), and syphilis, which were all negative. He had unprotected sex with multiple anonymous sex partners in the last 15 months after his compulsory isolation drug treatment, and his last unprotected casual sex was with a woman he met in a bar, which occurred 3 weeks before he visited our hospital.

On admission, physical examination revealed an infiltrative, hard erythematous lesion surrounding the coronal sulcus, with mild erosion and a small amount of exudation. There was a whitish pseudomembrane-like covering on the surface of the erythematous lesion in the coronal sulcus and the glans penis was not involved (Supplementary Figure 1). There were no other mucosal or skin lesions observed on the head, face, oropharynx, trunk, perianal, limbs, hands, and feet. The patient’s inguinal lymph nodes were normal. Considering the clinical manifestations of the patient’s lesion (erythematous lesion with mild erosion, a small amount of exudation, and the whitish covering) somewhat mimicked clinical characteristics of candidal balanitis, fungi microscopic examination was performed and showed a negative result. The patient then received the blood tests for STDs, including Treponema pallidum particle agglutination assay (TPPA), Rapid plasma reagin (RPR) assay, and Human Immunodeficiency Virus (HIV); Herpes simplex virus (HSV) type 1IgG, IgM, HSV type 2 IgG, IgM, which revealed positive in TPPA and HSV1, 2IgG test, and negative in RPR, HIV, HSV1, and 2 IgM tests.

Based on the fact that the patient had high-risk sexual behaviors 3 weeks before visiting our hospital, that he had an infiltrative, hard erythematous lesion surrounding the coronal sulcus hardly with any symptoms, and that he had no STDs history, a diagnosis of primary syphilis was made and a dose of benzylpenicillin 2.4 million units was given intramuscularly according to the guideline of the STD Association, China CDC (9). One day after the first dose of benzylpenicillin treatment, the patient came back to our hospital and reported that his mild burning sensation completely disappeared, and the hard erythematous lesion tended to soften. On examination, the redness and swelling of the erythematous lesion on the coronal margin subsided, and the exudation could not be observed. The whitish covering was significantly drier than that before treatment and the hard erythematous lesion seemed to soften (Supplementary Figure 2). After that, the patient returned to the hospital every day for another 6 days to have the above lesion observed. The erythematous lesion and whitish covering disappeared gradually, and the test for syphilis performed on day 7 after the treatment showed the result of TPPA (+) RPR (-). Then, on the 14th follow-up, the skin lesions of the coronal sulcus seemed to have almost disappeared without sequelae other than slight erythema at the site of previous lesions (Supplementary Figure 3). The blood TPPA and RPR was then rechecked and showed positive in TPPA and negative in RPR. Sixty days after treatment, the patient came back to the hospital again for the follow-up of the serum treponema and non-treponemal tests, which showed positive TPPA and RPR, and the RPR titer was 1:1. Dermatological examination showed that the skin of the coronal sulcus had completely returned to normal (Supplementary Figure 4). No other skin or mucosal lesions were observed, and the patient reported that he had no sex after syphilis treatment.

## Discussion

The syphilitic chancre is in the first stage of syphilis, appearing within 90 days from the Treponema pallidum inoculation (4). The typical syphilitic chancres could be a painless, hard, and erosive plaque or nodule or superficial ulceration with a clear margin, which is easily recognized. However, these typical clinical manifestations are seen only in about 40% of primary syphilis cases, and the remaining cases may be atypical, making the diagnosis difficult (7), especially when chancre is absent in primary syphilis.

Syphilitic balanitis of Follmann (SBF) is a rare condition of primary syphilis, which is characterized by any kind of balanitis, with or without chancre on the penis, as well as with the presence of swollen inguinal lymph nodes confirmed by the finding of Treponema pallidum in the lesions or by the positive serological syphilitic testing (8).

SBF was first described by Follmann, a dermatologist from Budapest. In 1931, 1934, and 1939 he declared that erosive balanitis could be the only manifestation of primary syphilis (10). Thereafter, the reported cases of SBF could be seen occasionally and the variable clinical manifestations of SBF were revealed. People believe that except for the absence of a chancre, SBF could be seen before, after, or at the same time as a chancre (11). SBF can present with any kind of balanitis, not just erosive balanitis.

Our case presented unusual clinical manifestations of SBF, the infiltration, erosion, exudation, pseudomembrane-like covering, and hard erythematous lesion were mainly seen in the coronal sulcus sparing the patient’s glans penis, and no enlargement of inguinal lymph nodes was observed, which were not consistent with the previous reports. Although the whitish covering on the surface of the coronal sulcus and the mild erosion of the erythematous base seemed somewhat like candidal infection, an infiltrative, hard erythematous lesion and the negative fungi microscopic examination refused the diagnosis of candidal balanitis. The reported case by Babu et al. described a patient who is HIV-positive with indurated SBF (12), and recently Mainetti et al. pointed out that many clinical manifestations are related to SBF, the skin lesions can be erosive or only indurated and scaly (8), which may partially support the viewpoint that our case is SBF. The short infection period of our case may explain why inguinal lymphadenopathy was not present. A suspicion of active herpetic infection could be made because of the positive serological tests of HSV IgG in our case. However, the patient reported that he had had no erosion or ulcer, or blister on his penis previously. The quickly subsided skin lesions after one dose of benzylpenicillin treatment, the positive serum treponema test, and the positive non-treponemal test could later be evidence of SBF diagnosis.

The diagnosis of primary syphilis could be a challenge in some cases. It is reported that 20–30% of the patients with chancre may have negative results in non-treponemal serological tests (13, 14). The positive serologic tests for Treponema pallidum appeared before non-Treponema pallidum. In our case, particularly, the short infection period of our case may explain why the non-treponemal serological test was negative before treatment. Then, the penicillin treatment further delayed the emergence of non-treponemal antibodies, which may explain why RPR was negative in the second week after treatment, and RPR turned positive 60 days after the treatment.

Since the patients refused to provide the skin samples, our case lacked etiological evidence, such as dark-field examination, histopathological findings, and PCR testing, which may limit its convincingness. However, this is a rare presentation of primary syphilis, which may help physicians to know more about the syphilitic balanitis of Follmann.

Our case suggests syphilitic balanitis of Follmann, which is a special type of primary syphilis that can have many different clinical manifestations and is easy to misdiagnose, especially when chancre is absent in this kind of primary syphilis. SBF can mimic any kind of balanitis, such as balanitis xerotica obliterans, plasma-cell balanitis, balanitis related to bacteria, virus, yeast, or parasite infection, and even trauma or irritants balanitis. Therefore, when balanitis with risky sexual activity or with other STDs, the diagnosis of SBF should be kept in mind.

## Conclusion

To our knowledge, this is the first SBF case reported in China. The variable clinical manifestations of syphilitic balanitis of Follmann indicate that SBF may not be as rare as believed. To exclude the diagnosis of SBF, a detailed sexual activity history should be investigated and Dark-field examination (DFE) or the polymerase chain reaction (PCR) swab testing for TP should be given, or serological tests for syphilis should be performed when patients are presented with any kind of balanitis.

## Data availability statement

The original contributions presented in this study are included in the article/Supplementary material, further inquiries can be directed to the corresponding author.

## Ethics statement

Ethical review and approval was not required for the study on human participants in accordance with the local legislation and institutional requirements. Written informed consent from the [patients/participants OR patients/participants legal guardian/next of kin] was not required to participate in this study in accordance with the national legislation and the institutional requirements.

## Author contributions

X-qR was responsible for receiving the patient, taking medical histories, comprehensively analyzing the patient’s condition, and diagnosing syphilitic balanitis of Follmann (SBF), which is a rare condition of primary syphilis and recorded the treatment process and followed up on the patient’s treatment. Q-lN collated in collaboration all the treatment records, pictures, and follow-ups. A-qL assisted in the diagnosis of the disease and provided advice on the treatment of the disease. All authors contributed to the article and approved the submitted version.
