# Supplementary material for: Primary syphilis without chancre – A case report of rare syphilitic balanitis of Follmann
Source: Front Med (Lausanne). 2022 Sep 21;9:958456. doi: 10.3389/fmed.2022.958456 (PMC9533062; doi:10.3389/fmed.2022.958456)

Supplementary Material

Supplementary Figure 1. Erythematous moist plaque surrounding the coronal sulcus of the patient's penis, with mild erosion, a small amount of exudation and whitish pseudomembrane-like covering (Before treatment.)

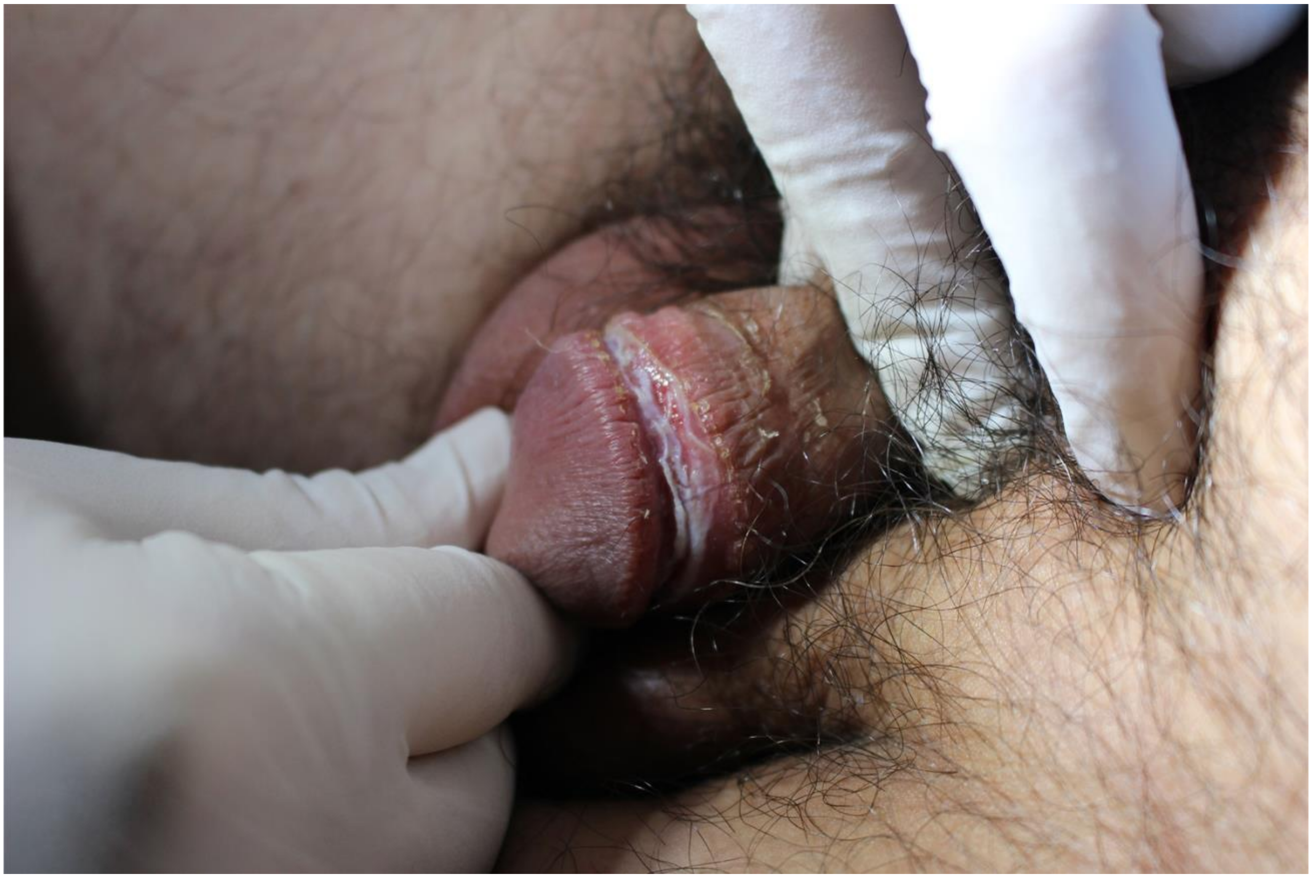

Supplementary Figure2. The redness and swelling of the erythematous lesion on the coronal margin subsided. The exudation resolved . The whitish covering became drier. (One day after treatment).

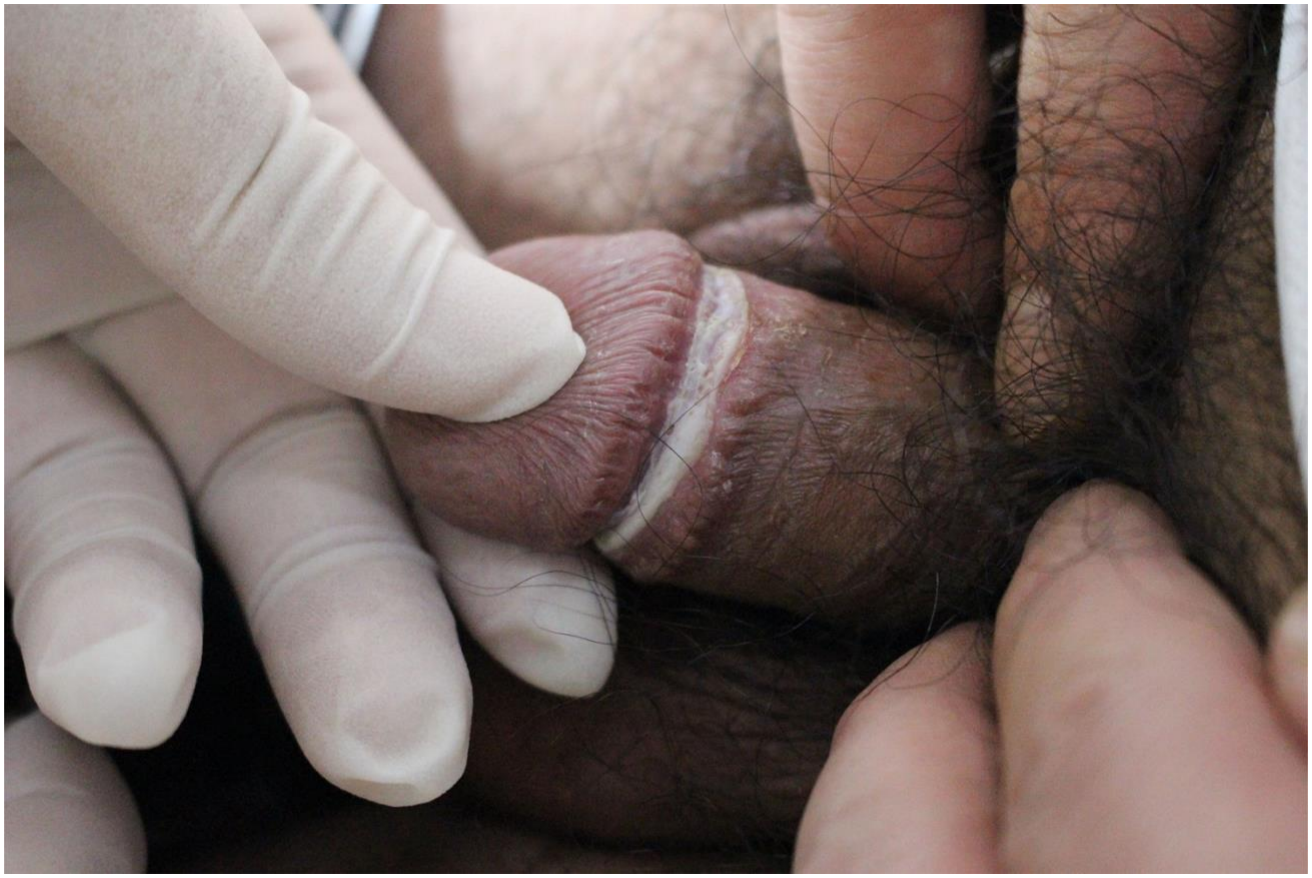

Supplementary Figure3 The skin lesions of the coronal sulcus almost resolved and left no sequelae. Mild redness was appreciated at site of previous". lesions. (Two weeks after treatment)

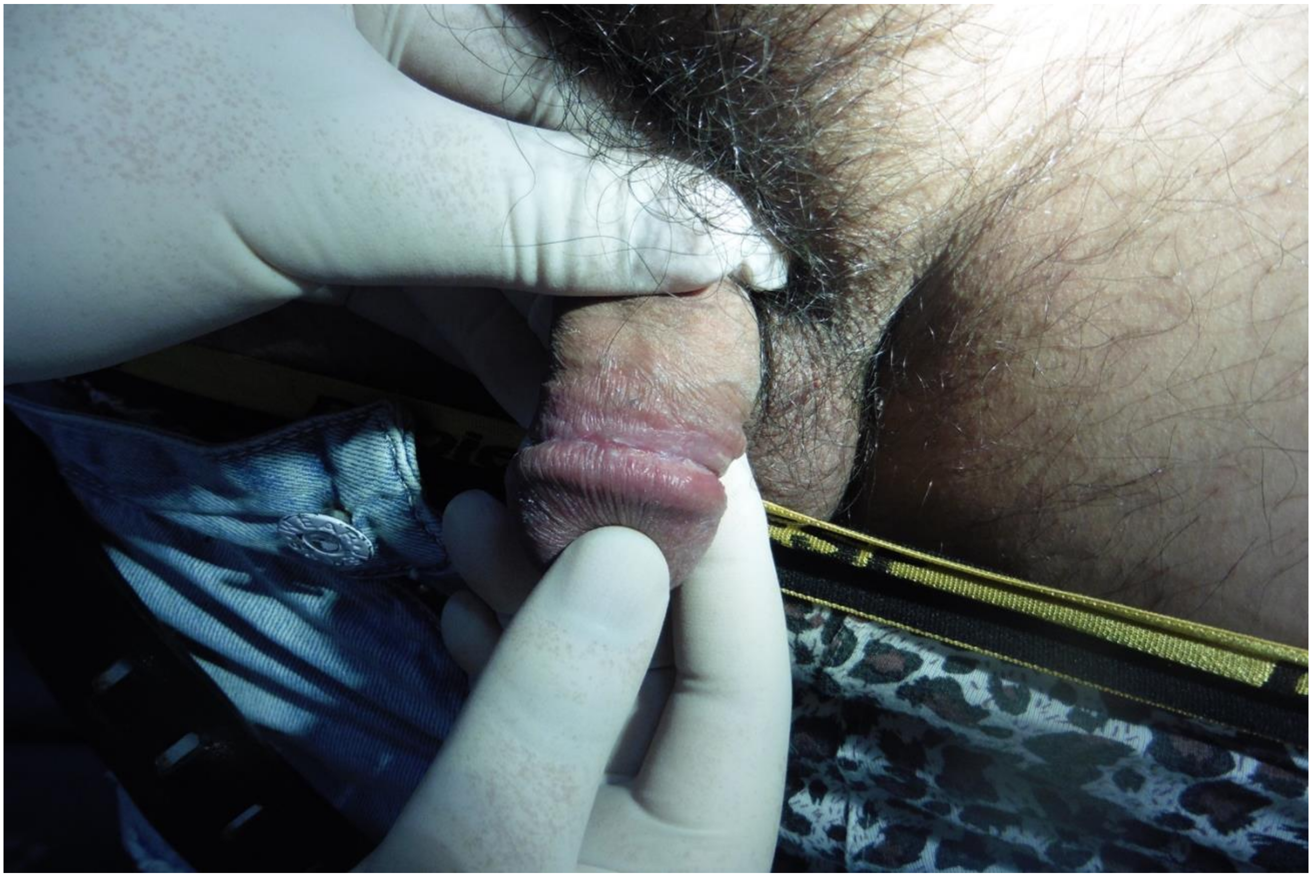

Supplementary Figure4 The normal skin on penis（60days after treatment）

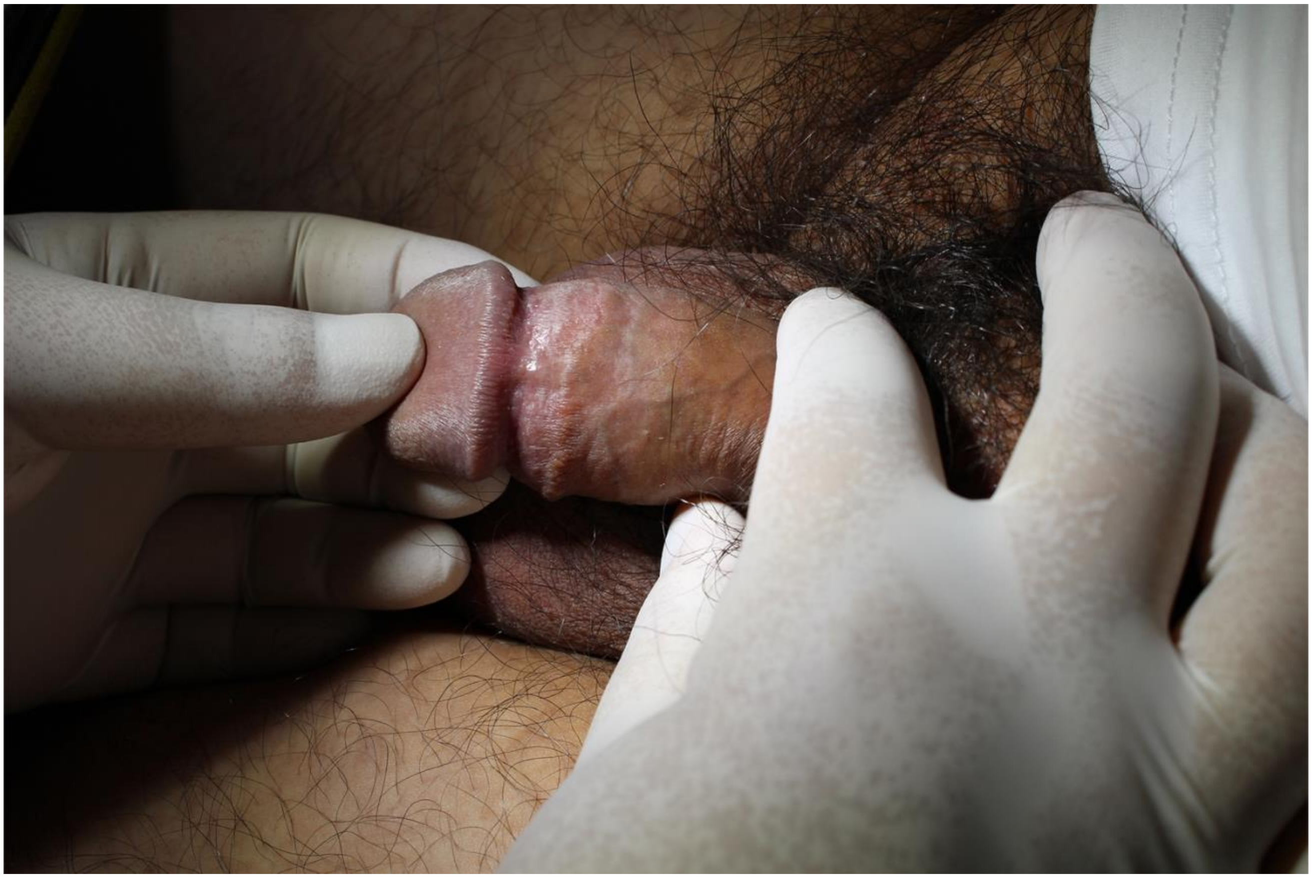

Supplement: Supplementary file 1 [file Data_Sheet_1.PDF]
